# Supplementary material for: I understand you feel that way, but I feel this way: the benefits of I-language and communicating perspective during conflict
Source: PeerJ. 2018 May 18;6:e4831. doi: 10.7717/peerj.4831 (PMC5961625; doi:10.7717/peerj.4831)
Supplement: Supplemental Information 1 — This supplementary file contains all the scenarios and statements that were used as part of this research. Participants rated each of the statements regarding the extent they believed the intended recipient would react in a defensive manner. [file peerj-06-4831-s001.docx]

Supplementary Material

For article: I understand you feel that way, but I feel this way: The benefits of I-language and communicating perspective during conflict, in *PeerJ*. Authors: Rogers, S. L., Howieson, J., & Neame, C.

**Scenario 1.**

*Ben and Jerry purchased a boat together, however at the time Jerry had something else come up and was not able to pay his half of the boat cost at the time of purchase. Ben had to pay the full price with the promise that he would be paid back in due course. A few months have passed and Ben is aware that Jerry is still not completely well off financially, but Jerry does now have the money to pay him back. Ben has expenses and could really use the money that is owed.*

# *In this section, a number of different statements are listed that Ben could use to begin a conversation with Jerry to resolve the issue. For each statement, choose from the options provided what you think the likelihood is that Jerry would respond in a defensive manner. In this survey the term 'defensive' refers to the other person reacting negatively or in a standoffish manner.*

Participants rated the statements below (presented in a random order), and for each statement were asked: *“What do you think is the likelihood that Jerry would respond defensively to the statement?”*, rating their response on a 6-point scale that ranged from ‘Not at all’ to ‘Extremely’.

The Statements:

Jerry, I understand that you are not completely well off financially, but I have expenses and could really use what is owed, can I get the boat-money back now?

Jerry, you are not completely well off financially, but I have expenses and could really use what you owe me, can you pay me the boat-money back now?

Jerry, I have expenses and could really use what is owed, can I get the boat-money back now?

Jerry, I have expenses and could really use what you owe me, can you pay me the boat-money back now?

Jerry, I understand that you are not completely well off financially, but can I get the boat-money back now?

Jerry, you are not completely well off financially, but can you pay me the boat-money back now?

Jerry, can I get the boat-money back now?

Jerry, can you pay me the boat-money back now?

**Scenario 2.**

*Carol and her work colleague Wendy have a joint project that has a submission deadline fast approaching. Both Carol and Wendy also have plenty of work to do on other individual projects. The joint project needs completing, however Wendy has expressed that she doesn’t really have the time to work on it because she has other things to do. Carol feels that it is unfair for her to do all the work on it by herself.*

*In this section, a number of different statements are listed that Carol could use to begin a conversation with Wendy to resolve the issue. For each statement, choose from the options provided what you think the likelihood is that Wendy would respond in a defensive manner to the statement. In this survey the term 'defensive' refers to the other person reacting negatively or in a standoffish manner.*

Participants rated the statements below (presented in a random order), and for each statement were asked: *“What do you think is the likelihood that Wendy would respond defensively to the statement?”* rating their response on a 6-point scale that ranged from ‘Not at all’ to ‘Extremely’.

1. Wendy, I understand that you are really busy, but I feel it is unfair that I have to do all the work on my own, and I think you should help on the project.
2. Wendy, you are really busy, but it is unfair that I have to do all the work on my own, and you should help on the project.
3. Wendy, I feel it is unfair that I have to do all the work on my own, and I think you should help on the project.
4. Wendy, it is unfair that I have to do all the work on my own, and you should help on the project.
5. Wendy, I understand that you are really busy, but I think you should help on the project.
6. Wendy, you are really busy, but you should help on the project.
7. Wendy, I think you should help on the project.
8. Wendy, you should help on the project.

**Scenario 3.**

*Mike and his partner Lucy are living together and both working full time. Whenever Mike does some cleaning of the house and asks Lucy to help, Lucy typically replies that she is too tired after a full day at work to do cleaning. Mike feels it is unfair that he should be responsible for all the cleaning duties.*

*In this section, a number of different statements are listed that Mike could use to begin a conversation with Lucy to resolve the issue. For each statement, choose from the options provided what you think the likelihood is that Lucy would respond in a defensive manner to the statement. In this survey the term 'defensive' refers to the other person reacting negatively or in a standoffish manner.*

Participants rated the statements below (presented in a random order), and for each statement were asked: *“What do you think is the likelihood that Lucy would respond defensively to the statement?”* rating their response on a 6-point scale that ranged from ‘Not at all’ to ‘Extremely’.

1. Lucy, I understand that you are very tired after work, but I feel it is unfair that I have to do all the cleaning by myself, and I think you should help with the cleaning.
2. Lucy, you are very tired after work, but it is unfair that I have to do all the cleaning by myself, and you should help with the cleaning.
3. Lucy, I feel that it is unfair that I have to do all the cleaning by myself, and I think you should help with the cleaning.
4. Lucy, it is unfair that I have to do all the cleaning by myself, and you should help with the cleaning.
5. Lucy, I understand that you are very tired after work, but I think you should help with the cleaning.
6. Lucy, you are very tired after work, but you should help with the cleaning.
7. Lucy, I think you should help with the cleaning.
8. Lucy, you should help with the cleaning.

**Scenario 4.**

*Susie and her friend Jane have been catching up every now and again over dinner at a local pub for a long time. However on the last few occasions Jane orders more drinks than usual and ends up becoming quite drunk. Susie feels confident that Jane is drinking heavily as a coping mechanism to deal with a relationship break up that occurred just before the increased drinking behaviour began. Susie is worried about her friend’s health.*

*In this section, a number of different statements are listed that Susie could use to begin a conversation with Jane to resolve the issue. For each statement, choose from the options provided what you think the likelihood is that Jane would respond in a defensive manner to the statement. In this survey the term 'defensive' refers to the other person reacting negatively or in a standoffish manner.*

Participants rated the statements below (presented in a random order), and for each statement were asked: *“What do you think is the likelihood that Jane would respond defensively to the statement?”* rating their response on a 6-point scale that ranged from ‘Not at all’ to ‘Extremely’.

1. Jane, I understand that you are hurting after the break up, but I feel that the extra alcohol is doing you no good, and I think the drinking should be slowed up a bit?
2. Jane, you are hurting after the break up, but the extra alcohol is doing you no good, and your drinking should be slowed up a bit?
3. Jane, I feel that the extra alcohol is doing you no good, and I think the drinking should be slowed up a bit?
4. Jane, the extra alcohol is doing you no good, and your drinking should be slowed up a bit?
5. Jane, I understand that you are hurting after the break up, but I think the drinking should be slowed up a bit?
6. Jane, you are hurting after the break up, but your drinking should be slowed up a bit?
7. Jane, I think the drinking should be slowed up a bit?
8. Jane, your drinking should be slowed up a bit?

**Scenario 5**

*Charlie has been designated a role as organiser of the monthly work meetings. Charlie feels the meetings work well to foster a sense of togetherness and collaboration amongst colleagues that helps maintain staff morale. However a newly appointed staff member, Brian, has been consistently not showing up to meetings. One of Charlie’s other colleagues has mentioned to him that Brian believes that since he is new he doesn’t have much say and so thinks it is pointless to attend meetings.*

*In this section, a number of different statements are listed that Charlie could use to begin a conversation with Brian to resolve the issue. For each statement, choose from the options provided what you think the likelihood is that Brian would respond in a defensive manner to the statement. In this survey the term 'defensive' refers to the other person reacting negatively or in a standoffish manner.*

Participants rated the statements below (presented in a random order), and for each statement were asked: *“What do you think is the likelihood that Brian would respond defensively to the statement?”* rating their response on a 6-point scale that ranged from ‘Not at all’ to ‘Extremely’.

1. Brian, I understand that you feel being new means you don’t have a great deal to contribute at the meetings, but I feel it is good for staff morale if we are all present, and I think you should attend staff meetings more regularly.
2. Brian, you feel being new means you don’t have a great deal to contribute at the meetings, but it is good for staff morale if we are all present, and you should attend staff meetings more regularly.
3. Brian, I feel it is good for staff morale if we are all present, and I think you should attend staff meetings more regularly.
4. Brian, it is good for staff morale if we are all present, and you should attend staff meetings more regularly.
5. Brian, I understand that you feel being new means you don’t have a great deal to contribute at the meetings, but I think you should attend staff meetings more regularly.
6. Brian, you feel being new means you don’t have a great deal to contribute at the meetings, but you should attend staff meetings more regularly.
7. Brian, I think you should attend staff meetings more regularly.
8. Brian, you should attend staff meetings more regularly.

**Scenario 6**
*Sharon is living with her partner Craig, and recently Sharon has noticed that Craig has been increasingly negative about things. If conversations don’t start out with a negative focus, then they typically end up becoming negatively focused by the end. Craig has been very busy and under a lot of pressure to perform at his work which he has mentioned has been getting to him. The constant negativity has been getting Sharon down and she feels the need to talk with Craig about it.*   

*In this section, a number of different statements are listed that Sharon could use to begin a conversation with Craig to resolve the issue. For each statement, choose from the options provided what you think the likelihood is that Craig would respond in a defensive manner to the statement. In this survey the term 'defensive' refers to the other person reacting negatively or in a standoffish manner.*

Participants rated the statements below (presented in a random order), and for each statement were asked: *“What do you think is the likelihood that Craig would respond defensively to the statement?”* rating their response on a 6-point scale that ranged from ‘Not at all’ to ‘Extremely’.

1. Craig, I understand that you have been feeling a lot of pressure at work lately and it is getting to you, but I feel the negativity is starting to bring me down too, so I think you should try to stop being so negative.
2. Craig, you have been feeling a lot of pressure at work lately and it is getting to you, but your negativity is starting to bring me down too, so you should try to stop being so negative.
3. Craig, I feel the negativity is starting to bring me down too, so I think you should try to stop being so negative.
4. Craig, your negativity is starting to bring me down too, so you should try to stop being so negative.
5. Craig, I understand that you have been feeling a lot of pressure at work lately and it is getting to you, but I think you should try to stop being so negative.
6. Craig, you have been feeling a lot of pressure at work lately and it is getting to you, but you should try to stop being so negative.
7. Craig, I think you should try to stop being so negative.
8. Craig, you should try to stop being so negative.
